# Extranodal Rosai-Dorfman disease involving the right atrium in a 60-year-old male

**DOI:** 10.1186/1746-1596-9-115

**Published:** 2014-06-09

**Authors:** Yalan Bi, Zhen Huo, Yunxiao Meng, Huanwen Wu, Jingbo Yan, Yuan Zhou, Xingrong Liu, Lan Song, Zhaohui Lu

**Affiliations:** 1 Department of Pathology, Peking Union Medical College Hospital, Chinese Academy of Medical Sciences and Peking Union Medical College, Beijing, China; 2 Department of Pathology, Xingtai People’s Hospital of Hebei Province, Hebei Province, China; 3 Department of Cardiac Surgery, Beijing Anzhen Hospital, Capital Medical University, Beijing, China; 4 Department of Cardiac Surgery, Peking Union Medical College Hospital, Chinese Academy of Medical Sciences and Peking Union Medical College, Beijing, China; 5 Department of Radiology, Peking Union Medical College Hospital, Chinese Academy of Medical Sciences and Peking Union Medical College, Beijing, China

**Keywords:** Rosai-Dorfman disease, Right atrium, Differential diagnosis

## Abstract

Rosai-Dorfman disease (RDD) involving the cardiovascular system is extremely rare; to our knowledge, there are only 9 cases in the literature. Here, a case of a 60-year-old male with RDD involving the right atrium is presented. A comprehensive literature review was undertaken to summarize the clinical and pathologic features of this disorder.

**Virtual Slides:**

The virtual slide(s) for this article can be found here: http://www.diagnosticpathology.diagnomx.eu/vs/2143194139120169.

## Letter to the editor

**Figure 1:**
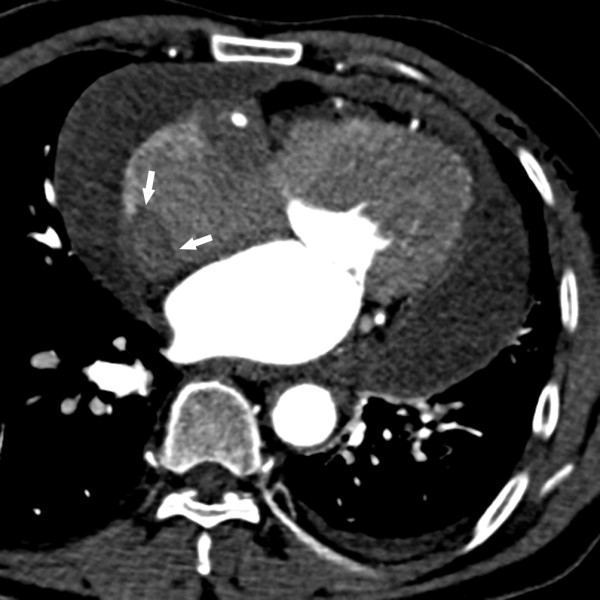
A contrast-enhanced computed tomography image demonstrating an irregularly shaped, 25.0-mm right atrial mass extending along the right atrial free wall (white arrow); minor left-sided pleural effusion; slight pleural thickening of the right side; and diffuse pericardial effusion.

**Figure 2:**
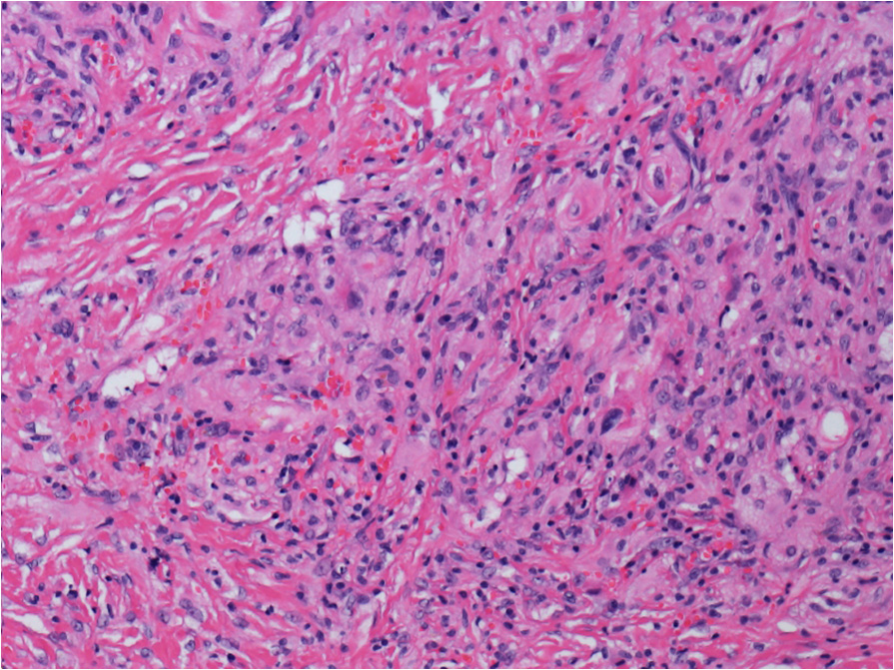
The tumor, showing histiocytic and chronic fibroinflammatory exudation, with areas of fibrosis (hematoxylin and eosin stain).

**Figure 3:**
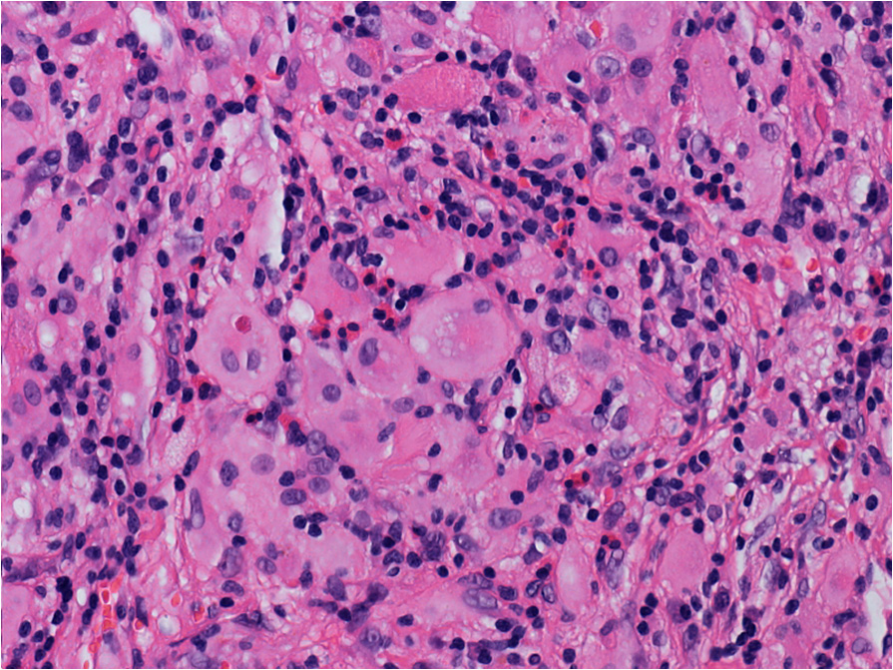
Histiocytes with large vesicular nuclei and abundant clear cytoplasm (hematoxylin and eosin stain).

**Figure 4:**
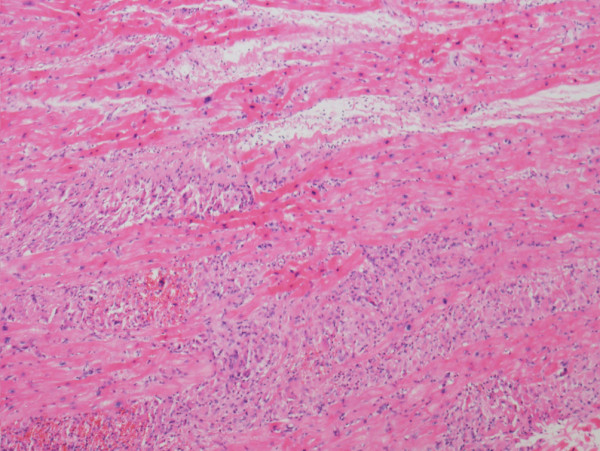
Diffuse involvement of the myocardial interstitium and the myocardium (hematoxylin and eosin stain).

**Figure 5:**
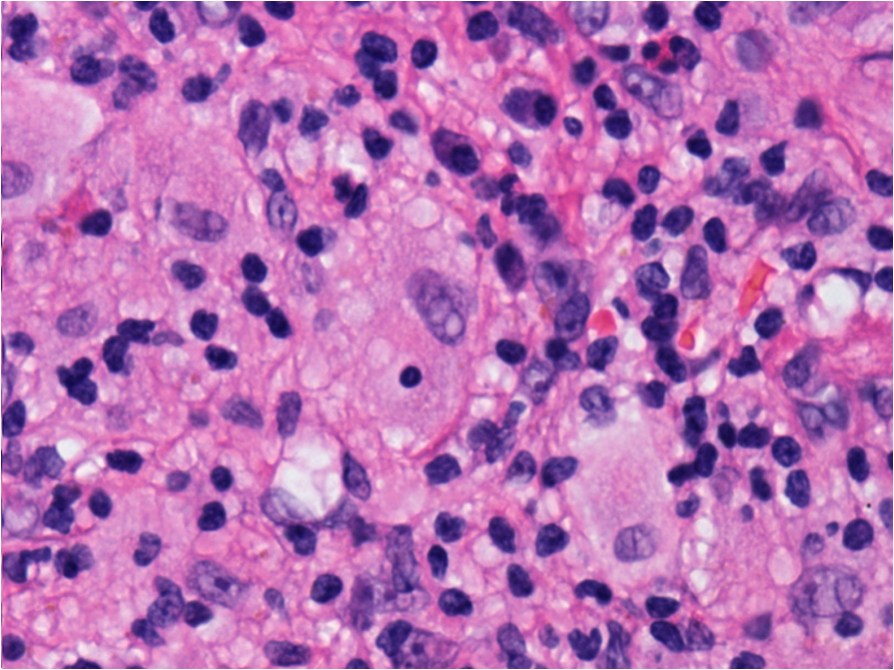
Histiocyte showing emperipolesis (hematoxylin and eosin stain).

**Figure 6:**
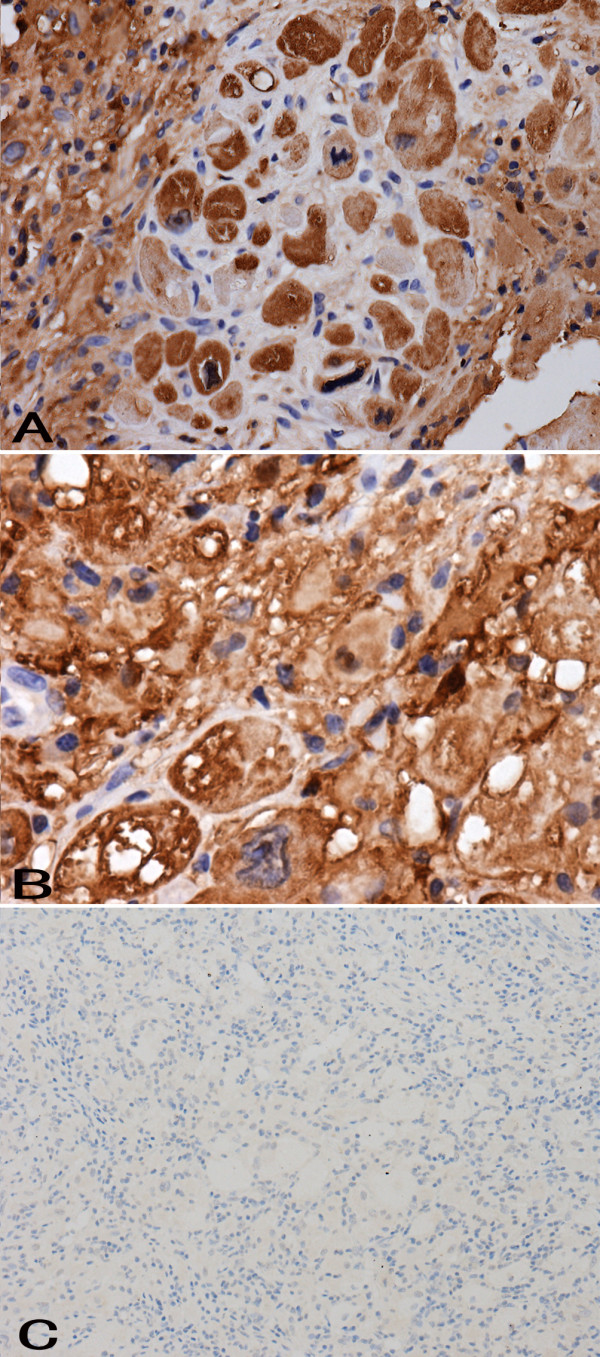
**Numerous histiocytes showing strong cytoplasmic immunohistochemical staining for S-100 (A).** Numerous histiocytes showing strong cytoplasmic immunohistochemical staining for CD68 **(B)**. Immunohistochemical staining showing CD1a negativity among histiocytes **(C)**.

Rosai-Dorfman disease (RDD), first reported by Rosai and Dorfman in 1969 and originally described as sinus histiocytosis with massive lymphadenopathy, presents in its most typical form as massive, painless bilateral lymph node enlargement in the neck. This symptom is associated with fever, leukocytosis, an elevated erythrocyte sedimentation rate, and polyclonal hypergammaglobulinemia [1]. RDD is a rare, idiopathic, non-neoplastic histiocytic disorder, and heart involvement is extremely rare. Only 9 cases involving the heart have been reported to date, 4 of which involved the atrium [2-4]. We here report one case of RDD involving the right atrium and provide a brief literature review, aiming to (i) avoid the trap of misdiagnosis of malignant or infectious diseases, (ii) emphasize the diagnostic histopathologic features of this disease, and (iii) achieve a better understanding of the character and treatment of this disease.A 60-year-old man presented with shortness of breath and fatigue. His medical history was remarkable for bilateral renal artery stenosis and meningioma. Comprehensive physical examination was unremarkable, except for a few premature heartbeats. Further evaluation, including an echocardiogram, revealed a cardiac mass involving the right atrium. A contrast computed tomographic scan of the chest showed a 2.5-cm irregular, poorly circumscribed mass with marked inhomogeneous enhancement in the right atrium, near the orifice of the superior vena cava (Figure 1). The patient underwent surgical resection of the portion of the right atrium with the attached atrial mass.Gross examination of the specimen revealed 2 pieces of an irregular, tan-white mass measuring 4 × 3 × 2 cm and 2 × 2 × 1 cm. The cut surface was tan-white to tan-yellow; fleshy, with focal fibrous areas; and poorly circumscribed. Microscopic examination of the hematoxylin and eosin (HE)-stained slides revealed exuberant histiocytic and chronic fibroinflammatory exudation, which was composed of numerous histiocytes with large vesicular nuclei and abundant clear or lightly eosinophilic cytoplasm, all with areas of fibrosis (Figure 2). Huge foamy xanthomatous histiocytes were common (Figure 3). There was diffuse myocardial interstitial involvement, with extension to the depth of the myocardium (Figure 4). Several few histiocytes had numerous intact lymphocytes within their cytoplasm, a feature that has been designated as emperipolesis or lymphocytophagocytosis. Although not specific, this phenomenon is a constant feature of RDD and is therefore of great diagnostic significance (Figure 5). Necrosis and significant mitotic activity were absent. Microorganisms were not identified by Gomori-Grocott methenamine silver (GMS), periodic acid-Schiff (PAS), or acid-fast staining. Immunohistochemical analysis revealed numerous histiocytes that were diffusely positive for S-100 and CD68 but not for CD1a (Figure 6). Staining for CD20 and CD3 showed a mixed population of B and T lymphocytes in the background. The plasma cells showed a polyclonal pattern of immunoglobulin expression. Immunostaining for IgG4 was negative in the plasma cells.

To date, approximately 7 months of routine follow-up after the surgery has been performed; the patient has not received any further treatment and remains well, with no recurrence.

The involvement of an extranodal site in association with lymphadenopathy is present in approximately 43% of all RDD cases, and only 23% of cases show isolated extranodal RDD [5]. The skin, soft tissue, and upper respiratory tract are the most frequently affected sites [5]. The 4 reported cases involving the right atrium included 2 males and 2 females ranging in age from 40 to 79 years. Two patients presented with chest pain, whereas the other two cases were incidentally diagnosed during autopsy. The sizes of the lesions ranged from 0.6 to 5 cm, showing variable numbers of pale-staining histiocytes with emperipolesis and a lymphoplasmacytic infiltrate on a background of fibrosis. Our case showed similar histologic findings associated with prominent fibrosis and rare emperipolesis. The histopathologic features of RDD at extranodal sites reflect the fact that fibrosis tends to be more pronounced and lymphocytophagocytosis less conspicuous than the nodal disease, which makes extranodal diseases more difficult to identify histopathologically. The immunohistochemical staining of lymph nodes and extranodal sites are similar to what has been previously reported. The immunohistochemical staining of our case showed S-100 and CD68 positivity in histiocytes, which is similar to the features of nodal disease. The lack of IgG4-positive plasma cells ruled out IgG4-related sclerosing disease. Of the two living patients with right atrial involvement who have been described in the literature, one took steroids, and both disclosed no evidence of disease progression. In most cases, RDD undergoes quick and complete spontaneous resolution. However, the involvement of vital organs can be associated with a potentially fatal outcome [6]. The prognosis of atrial RDD remains unclear because cardiovascular system involvement is extremely uncommon.

The differential diagnoses of a cardiac mass with microscopic features similar to those of RDD include Langerhans cell histiocytosis (in which the cells are positive for both S-100 protein and CD1a), an inflammatory myofibroblastic tumor (which has a background proliferation of spindle cells associated with an infiltrate of mononuclear inflammatory cells and ALK-1 positivity), metastatic malignant melanoma (in which cells are positive for Melan-A), Hodgkin’s disease (which shows characteristic Reed-Sternberg cells and positivity for CD15 and CD30), and fungal or mycobacterial infections (which are validated by positive staining with GMS, PAS, and acid-fast stain).

RDD is a rare entity, and RDD with cardiac involvement is extremely rare. Doppler echocardiography, computed tomography, and magnetic resonance imaging are useful in detecting and characterizing masses but are not specific for RDD. Pathologists must be aware that RDD should be considered among the possible differential diagnoses for cardiovascular tumors.

## Consent

Written informed consent was obtained from the patient prior to publication of this case report and the accompanying images. A copy of the written consent is available for review by the Editor-in-Chief of this journal.

## Competing interests

The authors declare that they have no competing interests.

## Authors’ contributions

YLB and ZH were the main authors on the paper, worked up the case, and drafted the manuscript. YXM and HWW participated in the design of the study. JBY conducted the immunohistochemical study. YZ was responsible for the clinical data. XRL was the surgeon who operated on the patient and who interpreted the patient’s data. LS participated in the radiological analysis. ZHL was the main pathologist involved in the case, made the final diagnosis, and was the main editor of the body of the text. All of the authors read and approved the final manuscript.
